# Platelet-Rich Plasma Applications for Achilles Tendon Repair: A Bridge between Biology and Surgery

**DOI:** 10.3390/ijms22020824

**Published:** 2021-01-15

**Authors:** Sabino Padilla, Mikel Sánchez, Victor Vaquerizo, Gerard A. Malanga, Nicolás Fiz, Juan Azofra, Christopher J. Rogers, Gonzalo Samitier, Steven Sampson, Roberto Seijas, Ricardo Elorriaga, Jack Taunton, Frank Boehm, Roberto Prado, Ramón Cugat, Eduardo Anitua

**Affiliations:** 1 Eduardo Anitua Foundation for Biomedical Research, Jacinto Quincoces, 39, 01007 Vitoria, Spain; roberto.prado@bti-implant.es; 2 BTI-Biotechnology Institute ImasD, Jacinto Quincoces, 39, 01007 Vitoria, Spain; 3 University Institute for Regenerative Medicine & Oral Implantology-UIRMI (UPV/EHU-Fundación Eduardo Anitua), Jacinto Quincoces, 39, 01007 Vitoria, Spain; 4 Arthroscopic Surgery Unit, Hospital Vithas Vitoria, Beato Tomás de Zumarraga 10, 01008 Vitoria-Gasteiz, Spain; mikel.sanchez@ucatrauma.com (M.S.); nicolas.fiz@ucatrauma.com (N.F.); juan.azofra@ucatrauma.com (J.A.); 5 Advanced Biological Therapy Unit, Hospital Vithas Vitoria, 01008 Vitoria-Gasteiz, Spain; 6 Department of Orthopaedic Surgery, Príncipe de Asturias University Hospital, 28805 Alcalá de Henares, Spain; vaquerizovictor@yahoo.es; 7 New Jersey Regenerative Institute LLC, Cedar Knolls, NJ 07927, USA; gmalangamd@hotmail.com; 8 Department of Physical Medicine and Rehabilitation, Rutgers School of Biomedical and Health Sciences, Newark, NJ 07901, USA; 9 Rutgers University and New Jersey Regenerative Medicine Institute, Cedar Knolls, NJ 07927, USA; 10 San Diego Orthobiologics Medical Group, Inc., Carlsbad, CA 92011, USA; rogers@sdomg.com; 11 Instituto Cugat, Plaza Alfonso Comín 5-7, Planta (-1), 08023 Barcelona, Spain; samitier@sportrauma.com (G.S.); seijastraumatologia@gmail.com (R.S.); ramon.cugat@sportrauma.com (R.C.); 12 Fundación García Cugat, c/Madrazo 43, 08006 Barcelona, Spain; 13 Hospital Quirónsalud Barcelona, Plaza Alfonso Comín 5, 08023 Barcelona, Spain; 14 David Geffen School of Medicine at UCLA, 10833 Le Conte Ave, Los Angeles, CA 90095, USA; drsampson@orthohealing.com; 15 The Orthohealing Center, 10780 Santa Monica Blvd, Suite 210, Los Angeles, CA 90025, USA; 16 Servicio de Cirugía Ortopédica y Traumatología, Hospital Universitario de Basurto, Montevideo Etorb, 18, 48013 Bilbao, Spain; rielorriaga@gmail.com; 17 Division of Sports Medicine, Allan McGavin Sports Medicine Centre, University of British Columbia, Chan Gunn Pavilion 2553 Wesbrook Mall, Vancouver, BC V6T 1Z3, Canada; jack.taunton@ubc.ca; 18 NanoApps Medical, Inc., Vancouver, BC V6K 1C8, Canada; frankboehm@nanoappsmedical.com

**Keywords:** Achilles, platelet-rich plasma, PRGF, tendon

## Abstract

Achilles tendon ruptures are very common tendon ruptures and their incidence is increasing in modern society, resulting in work incapacity and months off sport, which generate a need for accelerated and successful therapeutic repair strategy. Platelet-rich plasma (PRP) is emerging as adjuvant human blood-derived constructs to assist Achilles tendon rupture treatment. However, myriad PRP preparation methods in conjunction with poor standardization in the modalities of their applications impinge on the consistent effectiveness of clinical and structural outcomes regarding their therapeutic efficacy. The purpose of this review is to provide some light on the application of PRP for Achilles tendon ruptures. PRP has many characteristics that make it an attractive treatment. Elements such as the inclusion of leukocytes and erythrocytes within PRP, the absence of activation and activation ex vivo or in vivo, the modality of application, and the adjustment of PRP pH can influence the biology of the applied product and result in misleading therapeutic conclusions. The weakest points in demonstrating their consistent effectiveness are primarily the result of myriad PRP preparation methods and the poor standardization of modalities for their application. Selecting the right biological scaffold and applying it correctly to restitutio ad integrum of ruptured Achilles tendons remains a daunting and complex task.

## 1. Introduction

Achilles tendon rupture is a common tendon rupture and its incidence is increasing in modern society due to an aging population, the increasing prevalence of obesity, and expanded participation in sports [1]. Affecting the working population and recreational and professional athletes, Achilles tendon ruptures result in work incapacity and many months off sport, creating a significant requirement for accelerated and successful repair, while minimizing fibrosis and functional limitations [1,2,3]. Achilles tendinopathy is a painful and dysfunctional condition encompassing pathologies ranging from tendinosis or tendinitis to partial and full-thickness tears, where evidence is mounting that points to immunocompetent cells and activated stromal fibroblasts as the drivers of a non-resolved inflammatory condition [4,5,6,7]. Platelet-rich plasma (PRP) is emerging as an enhancer of the natural tendon healing process in the conservative and surgical treatment of Achilles tendon ruptures [2,3,8,9,10]. However, a lingering controversy exists as to whether these autologous blood-derived products are efficacious and facilitate expedited Achilles tendon repair [11,12,13,14,15]. The weakest points in demonstrating their consistent effectiveness are primarily the result of myriad PRP preparation methods and the poor standardization of modalities for their application. These elements somehow hamper advancement and lead to contradictory conclusions regarding their therapeutic efficacy [12,14,16].

This narrative review is intended to shed some light on the application of PRP for Achilles tendon ruptures. It endeavors to bring a number of pitfalls into focus regarding the varied methods used in PRP generation and the poor standardization of application modalities.

## 2. Aligning Coagulation and Hemostasis with Therapeutic Needs: PRP Products

Recent work toward deciphering the roles of blood components and of the biochemical machinery underlying the humoral and cellular arms of intravascular innate immune cascade systems, specifically coagulation and hemostasis as well as blood anticoagulation and deconstruction, have led to procedures that filter out platelets and the coagulation machinery to yield PRP [17]. These blood-derived products consist of fluid phase plasma, where platelets, white cells, and red cells are suspended in different concentrations, depending on the specific centrifugation and fractionation processes carried out [18].

### 2.1. When Fibrin Meets Growth Factors

**Figure 1:**
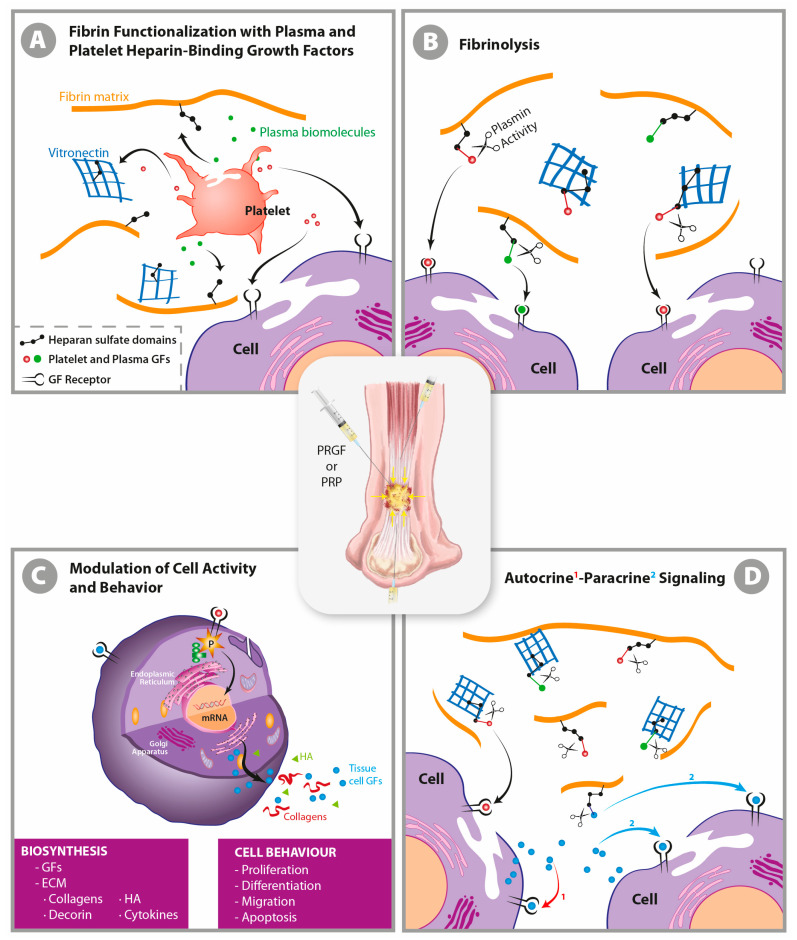
An autologous liquid-to-gel dynamic scaffold as a carrier of biological mediators in tissue repair. When liquid plasma rich in growth factors (GFs) is applied directly into tissue, the newly formed three-dimensional matrix clot traps many of the released growth factors and cytokines from platelet degranulation and from plasma by binding them to the heparin sulfate proteoglycan domains of fibrin matrix components in a non-diffusible mode (yuxtacrine or matricrine) (**A**). However, some GFs in a diffusion mode (autocrine and paracrine) will directly reach their cognate cell-surface receptor (**D**), thereby inducing an immediate cell-biosynthetic and cell-behavior modification (**C**). The ensuing progressive biodegradation of a fibrin clot is mediated by the serine protease plasmin, which is yielded through both the activation of plasminogen by a tissue plasminogen activator [24] and the immune and mesenchymal cells that migrate into the clot, thereby matching the speed of the ingrowing repair tissue [17,21,22,25,26] (**B**). GFs act as extracellular ligands by binding to transmembrane receptors arrayed on the surface of target cells, thereby activating intracellular signal transduction pathways that convey the signal to the nucleus to eventually induce a wide range of cell specifications during inflammation and the repair process including cell survival, proliferation, migration, differentiation, and maturation and changes in protein synthesis and metabolism (**C**). These effects include the synthesis and secretion of GFs and cytokines, which interact with their receptors in a diffusible manner (autocrine and paracrine pathways); the synthesis of extracellular matrix (ECM) components such as collagens, decorin, hyaluronic acid, and glycosaminoglycans; and cell survival, proliferation, differentiation, and migration (**C**) [17]. Only by understanding the unbreakable link between GFs and fibrin matrix will we grasp the in situ biological function of PRP and the additional pivotal fact that it is not necessary to combine this product with other delivery systems to slowdown the release of GFs.

When sodium citrate is used as an anticoagulant, the ex vivo recalcification of the fluid phase with CaCl_2_ reverts the coagulation cascade, which generates trace amounts of native thrombin that simultaneously induces the activation of platelets and their degranulation and the polymerization of plasma fibrinogen into a progressively functionalized insoluble fibrin matrix [17,19]. The newly functionalized three-dimensional fibrin nanoscaffold will bind in a non-diffusible manner and trap several platelet- and plasma-derived growth factors (GFs), chemokines, cytokines, and other biomolecules (TGFβ1 and β2, VEGF, PDGF, FGF, EGF, CTGF, SDF-1α HGF, IGF1, FXII, IL-8, and C3, among others) through the heparin sulfate proteoglycan domains of adhesive proteins, including fibrinogen, fibronectin (Fn), vitronectin (Vn), and TSP-1 [20,21,22,23]. Nevertheless, several GFs, in a diffusible mode, will directly connect with their cognate cell surface receptors (Figure 1A) to induce an immediate effect [22].

**Figure 2:**
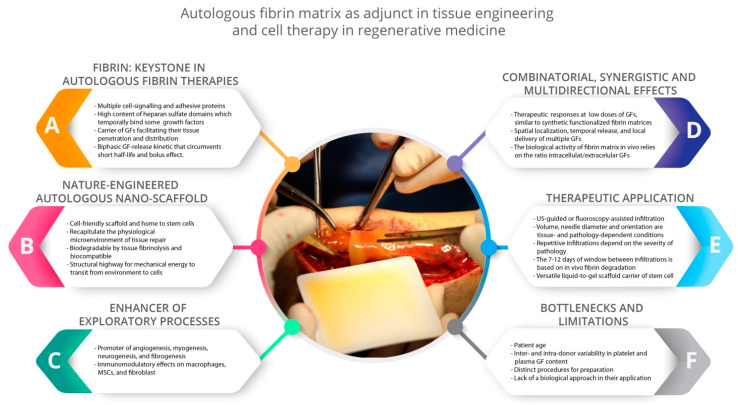
Summary of the primary biological and therapeutic features of platelet-rich plasma (PRP). PRP has emerged as an adjuvant human blood-derived constructs to assist tissue engineering and cell therapies in regenerative medicine. Figure adapted from [17] with permission.

In PRP, the architectural keystone is the fibrin matrix, as fibrin transiently binds several GFs, transports GFs to the vicinity of cells, and facilitates their tissue penetration, spatial localization, and in situ delivery (Figure 2) [17,21,27]. Moreover, fibrin circumvents the short GF half-life, and not only protects GFs from proteolysis, but also serves as a sink for morphogens and dampens the surge of GFs initially released by platelets (in conjunction with cytokines from leukocytes and the heme iron from erythrocytes, if present), thereby avoiding the bolus effect (Figure 2) [20,22,23,25,28]. Once an autologous fibrin scaffold has been applied, the breakdown of the matrix by tissue fibrinolysis (Figure 1B) releases GFs both immediately and in a gradual and delayed manner. Therefore, PRP operates as a biomimetic biphasic GF delivery systems, while modulating the spatiotemporal chemotactic gradients of the GFs required to induce cell–cell survival, migration, proliferation, differentiation, maturation, and correct orientation in nascent tissues (Figure 1B) [20,27]. The GFs released by platelets (among other tissue-resident cells of damaged areas) are biochemical signaling agents that operate on specifically limited length and time scales in autocrine and paracrine modes (Figure 1D). They diffuse over distances of less than several tens of micrometers; thus their signal intensity decays more than linearly with the distance from the source, roughly 1/r^2^; in 3D, it is even more rapid [29].

### 2.2. Tendon Tissue Effects of PRP: Multi-Directional Role of Growth Factors

PRP is a biodegradable autologous multi-growth factor scaffold product whose biological effect primarily relies on tissue-resident cells as the pivotal target of GFs conveyed by the fibrin liquid-to-gel dynamic scaffold. Ample evidence based on in vitro, in vivo, and clinical trials indicates that GFs, which are present within PRP and PRP products themselves, exert multi-directional biological effects on the mechanisms that govern tendon tissue repair, including (but not limited to) the following effects: angiogenic [30,31]; antiapoptotic, tenogenic, and proliferative [32,33,34]; chemotactic, trophic, and biosynthetic [9,30,31,32,33,34,35,36]; immunomodulatory and anti-inflammatory [34]; antifibrotic [3,30,31], antialgic [37,38]; and biomechanical [16,39,40]. If we focus on the effect of PRP on the extracellular matrix of rotator cuff tendons, Cross et al. [35] showed, in an elegant study, that leukocyte-free PRP stimulated normal collagen matrix synthesis (the ratio of COL1A1:COL3A1 gene expression) and decreased the cytokines associated with matrix degradation and inflammation, such as Matrix Metalloproteinases (MMP-9 and MMP-13) and IL-1β. These results were also confirmed by Yan et al. [36], demonstrating increased gene expression of Collagen 1 in PRP-treated Achilles tendons and decreased expression of catabolic proteins such as MMP-1 and MMP-3.

In the context of tissue development, regeneration, and repair processes, GFs behave in a combinatorial, synergistic, and multi-directional manner [41], which has inspired regenerative human-engineered approaches [21,22,41]. Accordingly, many successful strategies for therapeutic tissue regeneration and repair integrate a combination of several recombinant GFs that are frequently embedded in a fibrin scaffold [21,27], although in higher doses than the equivalent native growth factors present within autologous fibrin scaffolds [17]. Moreover, GFs within PRP fibrin matrices circumvent some of the drawbacks imposed by recombinant GFs, including their short half-life and bioavailability, high systemic toxicity, bolus effect, rapid cellular internalization rate, the limited spatial and time scales within which they operate, and the requirement of high therapeutic doses [22,28].

### 2.3. Practical Considerations in the Application of PRP for Achilles Tendon Ruptures

**Figure 3:**
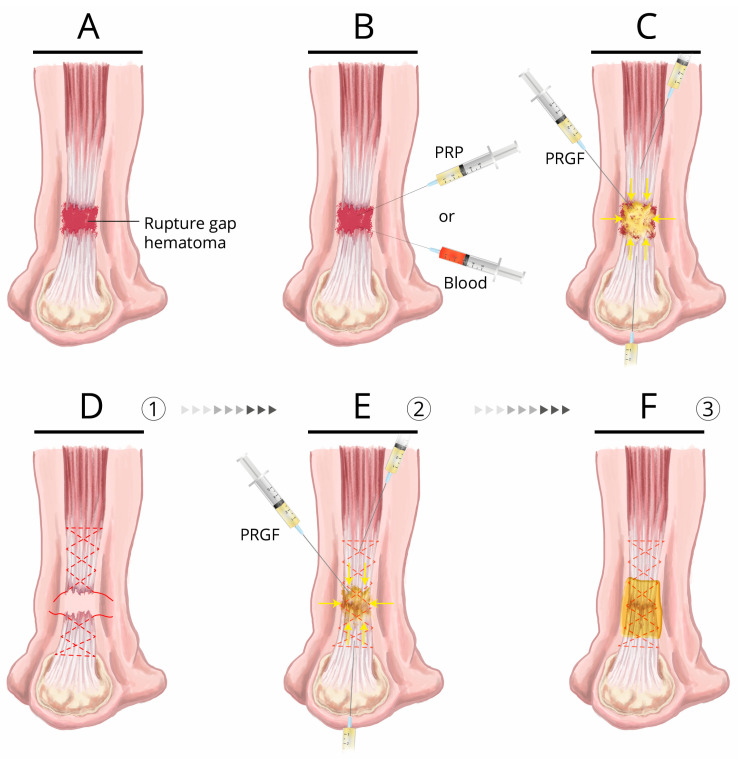
Achilles tendon rupture treatment: conservative and surgical treatment assisted by autologous blood or blood-derived products (PRP). (**A**) Achilles tendon rupture, rupture gap, and hematoma. (**B**) Conservative treatment: a single injection of either PRP or autologous blood injected into the gap hematoma. (**C**) Conservative treatment proposal (in patients with a low functional demand, or for whom surgery is contraindicated). Ultrasound-guided injection of 4 mL of liquid plasma rich in growth factors (PRGF) in close proximity to the apparently healthy tendon tissue at each stump of the tendon and 4–6 mL of liquid PRGF into the gap hematoma; a procedure to be repeated over 2 or 3 consecutive weeks (**D**–**F**) Surgical treatment proposal: after the suture is performed with a non-reabsorbable suture material that has been bathed in liquid PRGF (**D**), 4–6 mL of liquid PRGF is injected in close proximity to the apparently healthy tendon tissue at each stump, as well as into the sutured area (**E**) within the time window of 10 to 15 min following PRGF activation with CaCl_2_. This window encompasses the time when the fibrin scaffold is still macroscopically liquid is but undergoing microscopic jellification. Liquid PRGF can extensively permeate through areas that surround the injection site and anchor to the collagen and other ECM proteins exposed in damaged tissue margins through the activated platelets conveyed by the fibrin clot, as a 3D fibrin–extracellular matrix-like malleable structure [17], thereby bridging the gap of injured areas. The time window of 1–5 min is the result of the gel point or clotting time, meaning the change from liquid to solid undergone by the matrix when 15–20% of the fibrinogen has been incorporated into the gel by branching points (approximately 4 min 50 s) [46]. After closing the paratenon and prior to closing the overlying skin, the peritendinous regions are also infiltrated with PRGF; finally, the entire affected area of the tendon is covered with a fibrin membrane of PRGF (**F**). As general recommendation, we suggest the use of 10 mL Luer lock-type syringes with 21G needles, since the use of small syringes means that large pressures are exerted on the ECM of the tissue during infiltration, thereby accounting for the focalized disruption of the ECM components (a desirable effect in some treatment protocols aimed at disrupting the neovascularization and neoinnervation present in some chronic tendinopathies) [7,42]. Upon infiltration, the needle should be oriented as closely as possible parallel to, and longitudinal with, the tendon fascicles for an optimal diffusion of PRGF [42].

Our recommended conservative treatment of Achilles tendon ruptures (Figure 3A–C) usually begins with a dynamic ultrasound study, which should assess and verify that both ends of the rupture will approach each other during plantar flexion (evaluating their mobility), meaning that both extremes might heal effectively without excessive elongation [42]. Once the treatment strategy has been confirmed and is under ultrasound control, and with the foot plantar flexed, 6-8 mL of a liquid PRGF formulation is percutaneously infiltrated into the center of the rupture. In addition, 4 mL of liquid PRGF is infiltrated into each of the proximal and distal ends of the tendon (hyperechogenic ultrasound image of PRGF) [42] to recruit tendon and mesenchymal stem cells, as well as to target the stromal inflammatory microenvironment and stromal cell phenotypes, including macrophages and fibroblasts (Figure 3C) [5,43,44,45]. This infiltration process should be repeated over three consecutive weeks. The patient’s ankle is immobilized using a Walker-type orthosis in plantar flexion (equinus), which allows for partial and progressive loading. The equinus of the orthosis is progressively reduced from Week 4 onwards, removing a wedge every week until final removal of the orthosis. An ultrasound examination should be performed in Week 4 after the final infiltration. If unrepaired rupture regions are detected, the injured zone should be infiltrated again. Ultrasound examinations should be repeated every 4 weeks, together with dynamic testing until complete healing of the rupture is confirmed [42].

For the surgical treatment (recommended for recreational and professional athletes, for whom accelerated functional recovery is essential), the hematoma is evacuated, and the tendon edges are debrided. The tendon is sutured using a non-resorbable material, which has been previously soaked in PRGF, after which PRGF liquid is infiltrated into the sutured area, and the apparently healthy tendon of the proximal and distal stumps (Figure 3D,E) similar to the conservative treatment, and finally infiltrated into the peritendinous regions [3,42]. Following the closure of the paratenon and prior to closing the overlying skin, the affected area is covered with a PRGF membrane (Figure 3F). Subsequently, an ultrasound examination of the Achilles tendon is performed during the third week of recovery, the result of which might indicate PRGF infiltration. This proposed surgical treatment was initially implemented by Sanchez and colleagues in 2007 [3], who conducted a case–control study on athletes. They reported a faster recovery of motion, quicker resumption of sport activities, and a smaller increase in tendon cross-sectional area in contrast to a standard repair procedure after 18 months, a data compatible with a reduced fibrotic process in the PRGF group [3]. Other groups confirmed the histological and biomechanical beneficial effects of this procedure in both animals and humans [8,40]. Achieving a shorter immobilization time enables physiotherapy to be sped up due to the formation of a more efficient repair tissue. Therefore, for both the conservative and surgical treatment of Achilles tendon ruptures, early mechanotherapy and physiotherapy are of the utmost importance, and several studies revealed a synergistic effect between PRGF injections and early mechanical stimulation [3,47].

## 3. Pitfalls in the Application of PRP for the Repair of Achilles Tendon Ruptures

The weakest points in demonstrating the consistent effectiveness of PRP primarily reside in the myriad PRP preparation methods, in conjunction with poor standardization in the modalities of their application. These elements somehow hamper advancement and can lead to misleading conclusions regarding their therapeutic efficacy [12,16,17,18].

### 3.1. Whole Blood, Platelet-Rich Plasma, or Platelets Only?

Whole blood and PRP injections are used to treat Achilles tendon ruptures and tendinopathies with mixed clinical outcomes, which should be of no surprise due to both the different biological composition of PRP and the varied application modalities (Table 1) [3,6,8,10,11,12,13,14,38,48,49,50,51,52,53,54]. As an example of this heterogeneity regarding PRPs, Fitzpatrick et al. [55] conducted a meta-analysis of the effectiveness of PRP in the treatment of tendinopathies, which included heterogeneous blood-derived products from seven studies that used autologous whole blood, 11 studies that used leukocyte-rich PRP (LR-PRP), one study that used autologous conditioned serum, and one study that applied leukocyte-poor PRP (LP-PRP). PRP products are derived from widely varied methods of preparation [18] that generate a plasma fraction whose only common denominator is a platelet concentration that is superior to peripheral blood.

Furthermore, these methods eliminate, reduce, or concentrate white blood cells and erythrocytes in different proportions. These two factors will strongly impinge on the biological composition and therapeutic effectiveness of PRP from system to system [18,34,58].

In a randomized single-blinded study conducted on 30 patients who underwent the surgical repair of Achilles tendon rupture assisted by 10 mL of PRP injected into the rupture site, Schepull et al. [14] did not observe any biomechanical benefit assessed by elasticity modulus in Achilles tendon healing following rupture. However, Schepull et al. applied a platelet concentrate whose platelet concentration was about 17 times higher than the patient’s peripheral blood only into the rupture site [14]. The presence of plasma within PRP plays a critical role, since it conveys plasma GFs (HGF, IGF-1, FXII, and complement proteins, among others) and fibrin(ogen), whose absence renders the PRP procedure a bolus delivery system of only some growth factors. This may well prevent the penetration of GFs into tissues, as well as their spatial localization and delivery to the vicinity of cells; thus, exposing GFs to rapid proteolysis (Figure 2) [17,20,21,27].

Recently, Alviti et al. [10] conducted a retrospective trial of patients with acute Achilles tendon rupture who were surgically treated with or without LR-PRP matrices applied over the suture site, and reported an almost complete restoration of the biomechanics of the gait at 6 months in both groups. However, they found that the group of patients treated with PRP augmentation resulted in significant functional improvements in terms of ankle motion efficiency.

On the other hand, Keene et al. [12] conducted a multi-center randomized placebo-controlled trial that involved 113 patients with acute Achilles tendon rupture who were treated with a single percutaneous injection of 4 mL of LR-PRP into the center of the tendon gap following a previous injection of 1–2 mL local anesthetic into the skin area, avoiding anesthetics into the tendon itself (Figure 3B). The authors stated that the encouraging findings in laboratory studies with PRP did not translate into a detectable patient benefit in tendon injury healing, despite the laboratory studies not recommending the use of leukocyte PRP for the healing of injured tendons [12,34]. These and other results (Table 1) contrast with those obtained by Sanchez et al. [3] (the surgical treatment proposal in Figure 3D–F) and Arriaza et al. [8], which ultimately fueled the lingering controversy surrounding whether the inclusion of leukocytes (and erythrocytes) as key ingredients is required to achieve perfect clinical and structural outcomes in tendon repair and application protocols. In this respect, the majority of experimental research suggests that white blood cells (WBCs) exert catabolic and pro-inflammatory effects on tenocytes [28,34,35,36,59,60]. However, the clinical outcomes of PRP injections in tendinopathies are more heterogeneous, with some trials showing beneficial effects of LR-PRP injections compared with saline, corticoid, or control treatments [48,49,51,52,61], whereas other studies reporting no beneficial effects of LR-PRP or whole blood [6,12,38,50,53,54,62,63,64,65]. These conflicting results might be partially explained by the heterogeneity of tendinopathies; the anticoagulation, activation, administration, and rehabilitation methods; the type of tendon (Achilles, patella, supraspinatus, extensor carpi radialis brevis, and gluteus medius and minimus tendons); the number of injections; and patient age [66]. Moreover, recent data on tendon-derived stromal fibroblasts and macrophages from patients with Achilles tendinopathy, Achilles rupture, and diseased human supraspinatus tendons have suggested that these cells exhibit complex inflammation signatures involving the NF-κB pathway [5,43], which adds another layer of complexity to the pervasive yet incomplete degenerative paradigm of tendinopathies and tendon ruptures. Tendinopathic fibroblasts mount stronger inflammatory responses in vitro than those of healthy hamstring tendon tenocytes when exposed to IL-1β and IFNγ [5,43]; a fact associated with altered responsiveness of NF-κB [5,67]. Conceptualized as “stromal fibroblast memory” by Dakin [5,67], this new paradigm might partially account for the lack of improvement in pain and other clinical and functional outcomes of LR-PRP on ruptured and tendinopathic Achilles tendon, rotator cuff tendinopathies and tears, or even knee osteoarthritis, compared with placebo or saline treatments. This is likely to be partially derived from the pro-inflammatory and catabolic-induced effects of leukocytes on inflamed stromal cells [6,12,43,50,54,63,64,65,68]. Accordingly, injected leukocytes may induce the release of pro-inflammatory cytokines from primed stromal fibroblasts, which together with the detrimental effects of erythrocyte-derived heme-iron through pro-inflammatory macrophage polarization [44,58,68,69], may operate as non-resolving inflammation and profibrotic agents, respectively [5,7,67,68], even exacerbating the inflammation-driven tendinopathy [64,65]. In support of this, studies on tendinopathic fibroblasts, osteoarthritis synoviocytes, and tendon stem cells cultured in leukocyte-containing PRP supernatant have been reported to release significantly higher levels of several pro-inflammatory cytokines compared with leukocyte-depleted PRP [34,35,36], similar to PRP with leukocytes that simulate the inflammatory conditions with lipopolysaccharides (LPS) and whose supernatant releases significantly higher amounts of pro-inflammatory cytokines compared with the supernatant of LP-PRP [59]. Finally, platelets are an important source of Lipoxin A4 (LXA_4_), an endogenous arachidonic acid-derived pro-resolving mediator that has been reported to counter-regulate inflammatory processes in cells from patients with Achilles ruptures and Achilles tendinopathies [67].

### 3.2. PRP Application Modalities

Another important factor in the therapeutic application outcomes of PRP relates to the protocols utilized for its administration [17]. In a study by Keene et al. [12], the only percutaneous injection of non-activated PRP was infiltrated into the center of the Achilles tendon gap. Similarly, Schepull et al. [14] only applied a platelet concentrate into the rupture site following suturing. Alviti et al. [10] applied a platelet-rich fibrin (PRF) membrane but did not infiltrate the tendon stumps, yet significant functional improvements resulted in terms of the efficiency of ankle motion, contrary to Zou et al. [13], who injected into the tendon stumps but did not place a membrane, yet found biomechanical improvement over the short and midterm compared with a control group, but not at 2 years of follow-up (Table 1). On the other hand, Arriaza et al. [8] treated eight patients with chronic Achilles tears using a quadriceps tendon autograft, which was infiltrated with plasma rich in growth factors (PRGF), as well as the Achilles tendon stumps, and reported excellent clinical and functional scores. 

Tendon rupture sites present a gap that is usually occupied by a hematoma and necrotic tendon (Figure 3A) tissue that, in Achilles tendon ruptures treated surgically, are evacuated and debrided, respectively [42]. The ruptured areas are a source of inflammatory mediators which stem primarily from blood-derived neutrophils and monocytes, tissue-resident macrophages, erythrocyte-derived heme iron, and dying tenocytes [68]. In this context, PRP injected into the ruptured gap might exert anti-inflammatory, immunomodulatory, anti-apoptotic, and anti-fibrotic effects, provided that the PRP composition is leukocyte- and erythrocyte-free [3,34]. However, we suggest that to promote the functional and structural healing process of a ruptured tendon it is not enough to simply infiltrate PRP into the tendon gap. Further to the immunocompetent cells that reside in a healthy and dysregulated tissues next to the damaged region [43], another important target for the GFs and cytokines of PRP are the healthy tendon-residing stem cells and their niches, at both the healthy proximal and distal stumps of the ruptured tendon [3,70]. These should also be injected to stimulate tendon regrowth toward partially bridging the tendon gap (Figure 3C–E) [3]. Even in the best-case scenario, the function of the tendon–muscle unit would be biomechanically compromised due to the elongation generated by the newly formed tissue that bridges the tendon gap. Only by hypothesizing that tendon repair would be specifically driven through the adaptive cellular reprogramming of adult differentiated cells like tenocytes that survive within the hypoxic and necrotic tissue-injured microenvironment would the tendon heal through this single injection approach and therefore achieve the therapeutic purpose. There are several examples of this cell plasticity in tissue repair and regeneration including fibroblast/myofibroblast differentiation, Myelin/Remak Schwann cells repairing (Bungner) Schwann cell trans-differentiation, and macrophage (M1, M2, M3, M4) and neutrophil polarization [71,72], to which PRP might contribute as well [45,73,74,75].

### 3.3. PRP Activation and pH

Whereas ex vivo PRP activation with CaCl_2_ gives rise to a gradual liquid-to-gel dynamic scaffold and a functionalized fibrin matrix, which circumvents the short half-life and the rapid GF proteolytic degradation [17,19], the direct injection of non-activated PRP [11,12] conveys platelets that might interact with the injured tissue microenvironment. Whereas some platelets will be activated by tissue collagen–von Willebrand factor complexes, thrombin, and platelet-derived thromboxane A_2_ within the injured tissue [76] and release their cargo in a bolus manner, other platelets might adhere to monocytes and, through the nuclear translocation of NF-κB, induce the expression of NF-κB-dependent inflammatory genes on monocytes. In doing so, monocytes generate pro-inflammatory cytokines [77]. Furthermore, when directly injected non-activated PRP, platelets might activate the complement system and promote the formation of platelet–leukocyte complexes and neutrophil extracellular traps (NETs) in situ. This may exacerbate the already inflamed microenvironment, which may be amplified by the leukocytes of LR-PRP, as stated above [68,77]. On the other hand, there are doubts as to whether the in vitro determination of growth factors following the freezing of platelets and lysing them with Triton-X-100 [12] might correspond to growth factors released in vivo following contact with tendon collagen. Perhaps an in vitro activation with collagen would be more representative, since platelets respond differently to distinct types of stimulus. The additional activation of PRP with thrombin appears to be unnecessary, as CaCl_2_ adds calcium (Factor IV of the coagulation cascade), which restores extracellular matrix (ECM) calcium homeostasis and generates a low yet efficient amount of autologous thrombin [76], thereby yielding a gradual and dynamic liquid-to gel scaffold injectable within a roughly 1–5 min window, during which PRP is macroscopically manageable as a liquid, or a fibrin membrane 20 min following activation [3,17,19,23,42]. However, whereas low concentrations of thrombin exhibit growth factor-like fibroblast and endothelial cell proliferation migration, antiapoptotic, and inflammatory modulation activities, high doses of thrombin would operate as an inflammatory mediator that recruits monocytes, activates the NF-κB of endothelial cells, and triggers the release of cytokines from mast cells [24,76]. Moreover, exogenous activation with commercially available thrombin (bovine) might be associated with adverse effects, which include immune reactions, thrombosis, and hemorrhages [78].

Another area of concern relates to the pH of PRP, where PRP preparation devices and studies should provide its precise characterization, which should also include the pH of the product that is ready to be injected, among many other parameters that convey its biological properties [18,35]. In some studies, based on the acidic properties of acid citrate dextrose (ACD), which is used as anticoagulant, a basic buffer solution of 8.4% sodium bicarbonate was added to adjust an unknown PRP pH to physiologic pH prior to injection to address Achilles or tennis elbow tendinopathy [38,53,54,79]. As examples, the excellently designed and carefully conducted trials by Krogh et al. [53,54], who, through the use of an ultrasound-guided single percutaneous injection of LR-PRP into the elbow lateral common tendon and Achilles tendon, respectively, did not observe any clinical or functional improvement compared with a saline or glucocorticoid group. It is worthy of note that the acidity of plasma is contingent on the quantity of citrate used [79]. Some PRP devices may or may not use ACD as an anticoagulant (PRP pH values of 7.0 and 8.0 respectively) and, due to its acidic pH (ACD presents a pH=4.98 [35]), device manufacturers recommend the use of 8.4% sodium bicarbonate in the former procedure (Biomet GPS III, Biomet Biologics). Similarly, the pH values of other commercial PRP kits using ACD ranged from 6.59 (SmartPrep2, Terumo Harvest) to 7.05 (GPS III, Biomet Biologics) in the measures reported by Fitzpatrick et al. [80]. Accordingly, in a technical analysis of PRP applied to treat tendinopathies, Kaux et al. [81] reported that in the majority of studies that used ACD as an anticoagulant, researchers added a buffer solution. However, other devices that also use citrate (trisodium citrate) [79] present a pH of 7.64 ± 0.09 prior to activation and 7.53 ± 0.09 once it has been activated with 10% CaCl_2_ [82]. These pH values are far different from the acidic pH values reported by some studies and are attributed to CaCl_2_ (pH of 6.3) [18,78], which makes the addition of a buffer solution unnecessary in this case [82]. Importantly, the associated administration of local anesthetics (bupivacaine and lidocaine) and corticoids (methylprednisolone) with PRP should be avoided, since it has been reported that these drugs decrease the viability and proliferation of tenocytes [38,83,84].

## 4. Future Perspectives

Selecting the right biological scaffold and applying it correctly to restitutio ad integrum of ruptured Achilles tendons remains a daunting and complex task. Although PRP has many characteristics that make it an attractive treatment, its application is still nascent. The scientific community continues to learn and garner valuable insights to enhance the potential of PRP treatment via intellectual, technological, and research efforts into some of the biological and practical bottlenecks. Prior use and research of PRP has failed to fully address issues to optimize structural and functional PRP-mediated tendon repair outcomes; therefore, the clinical use of and research into PRP in the treatment of Achilles tendon tears has now been limited to its use as adjunctive treatment with surgery. The advances that emerge from synergies between natural and synthetic biology will lead to the generation of optimal biological scaffolds and guide their best possible application in the treatment of Achilles tendons in conjunction with functional rehabilitation.

## Figures and Tables

**Table 1:** Summary of studies on human Achilles tendon rupture treatment assisted with platelet-rich plasma.

Study	Features of the Study	PRP Characteristics	Modality of Application	Outcome
Sanchez et al. [3]	Retrospective case–control PRGF and surgery *n* = 6 Control *n* = 6 only surgery	Citrate as anticoagulant Activation with CaCl_2_ Leukocyte- and erythrocyte-free Platelets 2–3×	Injection of 4 mL of PRGF into the suture and surrounding areas. A PRGF membrane to cover the rupture and sutured area	Faster recovery of motion, quicker return to sporting activities, and a smaller increase in cross-sectional area on tendons treated with PRGF after 18 months
Sanchez et al. [56]	Case study *n* = 2 Surgical repair	Citrate as anticoagulant Activation with CaCl_2_ Leukocyte-and erythrocyte-free Platelets 2–3×	Infiltration of 3 mL of PRGF into each tendon stump. A PRGF membrane covering the affected areas	Successful PRGF-assisted management and recovery of major post-operative Achilles tendon infection and necrosis after primary surgical repair.
Schepull et al. [14]	Randomized controlled trial PRP *n* = 16 Control *n* = 14 Surgical repair	Citrate phosphate dextrose as anticoagulant Double centrifugation Platelet concentrate Activation with CaCl_2_ White blood cells (WBC) unreported Platelets 10–17×	6 mL of PRP into the ruptured site through a cannula and 4 mL of PRP transdermally in the ruptured site.	No beneficial effect of platelet concentrate addition in terms of the biomechanical properties of the tendon assessed by elasticity modulus. Detrimental effect of PRP compared with control on Achilles tendon total rupture scores at 12 months.
Alsousou et al. [9]	Conservative treatment Immunohistochemical study PRP *n* = 10	Unreported PRP system	Locally applied PRP.	PRP promotes better Collagen I deposition, decreased cellularity, less vascularity, and higher glycosaminoglycans (GAGs) content compared with the control.
Keene et al. [12]	Randomized placebo-controlled trial PRP 113 *n* = 10 Control *n* = 116 Conservative treatment	Leukocyte-rich (LR) PRP No activation Not ultrasound-guided (US)-guided WBC 2.2× Erythrocytes Platelets 4.1×	Local anesthetic injection, then single percutaneous non-US-guided injection of 4 mL PRP into the center of the tendon gap	No functional or clinical benefit in terms of muscle tendon maximum work, limb symmetry index, heel rise endurance test, pain, and adverse effects of PRP injection compared with the placebo.
De Carli et al. [11]	Case series PRP and surgery *n* = 15 Control, surgery only *n* = 15	Leukocyte-poor (LP) PRP Liquid PRP, no activation Gel PRP, activation with thrombin and Ca-gluconate Platelets 2–3×	Addition of 2 mL of liquid PRP near the sutured tendon 2 mL of gelatinized PRP sutured to paratenon. A second injection of 4 mL of PRP 14 days post-operatively	The addition of PRP to the surgery did not offer superior clinical and functional outcomes in terms of Visual Analogue Scale (VAS) and isokinetic ankle plantar and dorsal flexor range of motion compared with the control at 6 and 24 months.
Alviti et al. [10]	Retrospective comparative study Platelet-rich fibrin (PRF) *n* = 11 No PRF *n* = 9 Healthy *n* = 8 Surgical repair	LR-PRP Activation with batroxobin and Ca-gluconate to generate a membrane	Application of fibrin glue over the sutured site	Almost complete restoration of the biomechanics of the gait at 6 months independently of the use of PRF or not. The PRF group showed significant improvement in efficacy of motion
Zou et al. [13]	Prospective randomized trial PRP *n* = 16 Control *n* = 20 Surgical repair	LR-PRP WBC 4× Platelets 6×	Injection of PRP into the paratenon sheath and the surrounding lacerated tissue	PRP group showed significant short and midterm improvement in the ankle range of motion, pain, stiffness, and subjective scores (Leppilahti). At 2 years of follow-up, there were no differences between the PRP and control group for these parameters.
Arriaza at et al. [8]	Case series *n* = 8 Surgical reconstruction	Citrate as anticoagulant Activation with CaCl_2_ Leukocyte-and erythrocyte-free Platelets 2–3×	PRGF injections into the quadriceps autograft as well as into the tendon stumps	Successful repair of neglected chronic Achilles tears with significant improvement in AOFAS score and Boyden functional score after 2 years of follow-up.
Kaniki et al. [15]	Retrospective comparative study PRP *n* = 73 Control *n* = 72 Conservative treatment	LP-PRP No activation Lidocaine as anesthetic Not US-guided Platelets 2–3×	One injection of 3–4 mL of PRP into the deep gap that was repeated 2 weeks afterwards with the same protocol	No measurable benefit of the addition of PRP to the conservative treatment in terms of strength or range of motion of the ankle.
Boesen et al. [57]	Randomized double-blinded prospective study PRP = 19 Placebo = 19 Conservative treatment	LP-PRP No activation US-guided Platelets 2–3× (2.5× in one sample)	One injection of PRP (4 mL) into the rupture gap that was repeated every 2 weeks to complete a total of four injections	No differences between placebo and PRP groups in term of clinical benefits assessed by ATRS score or functional outcomes at any point of follow-up (2, 3, 4.5, 6, 9, and 12 months)

## Data Availability

Not applicable.
